# Effect of three different grafting materials on immediate implant placement using vestibular socket therapy in class II extraction sockets in the maxillary esthetic zone: a randomized controlled clinical trial

**DOI:** 10.1186/s12903-023-03345-9

**Published:** 2023-09-01

**Authors:** Mohamed Mofreh Hamed, Maher Mohamed El-Tonsy, Abdelsalam Elaskary, Gaser O. Abdelaziz, Safinaz Saleh Saeed, Bassem Nabil Elfahl

**Affiliations:** 1 Department of Oral Medicine, Oral Diagnosis & Radiology, Tanta University, Periodontology, Tanta, Egypt; 2 Department of Periodontology, Faculty of Dentistry, Alexandria University, Alexandria, Egypt

**Keywords:** Immediate implant, Facial bone plate, Fresh extraction sockets, Esthetic zone

## Abstract

**Background:**

This study compared the effectiveness of three bone grafting materials used for treating class II fresh extraction sockets in the esthetic zone with immediate implant placement using Vestibular Socket Therapy (VST) to evaluate the pink esthetic score (PES), peri-implant mucosal levels (PML), and facial bone thickness (FBT).

**Methods:**

Twenty-four surgical sites in the maxillary anterior region presented with type II socket defects received immediate implants and simultaneous bone grafting with either a collagen plug soaked in blood, demineralized bone matrix Grafton, or a particulate mixture of 2/3 autogenous bone chips and 1/3 deproteinized bovine bone mineral MinerOss X. The outcome measures were evaluated at 6 and 12 months. The study was registered on www.clinicaltrial.gov (**12/07/2021** - **ID: NCT04957654).**

**Results:**

Twenty-two cases (91.6%) showed a total PES score of > 10, without a significant difference between all groups. The vertical height soft tissue changes showed significant improvement in the Collagen plug and Grafton groups at 6 and 12 months, while MinerOss X showed no significant difference at 6 and 12 months compared to baseline. Radiographically, FBT was 0.72 ± 0.20, 0.44 ± 0.12, and 0.95 ± 0.37 at baseline, which significantly increased to 1.61 ± 0.88, 1.48 ± 1.20 and 2.31 ± 0.86 at 12 months for all three groups, respectively.

**Conclusion:**

The use of a particulate bone graft mixture significantly increases the FBT compared to collagen plugs and DBM-Grafton when performing VST during immediate implant placement in compromised Class II extraction sockets.

## Background

Immediate replacement of teeth with dental implants was proposed several decades ago. It minimizes the treatment time between tooth extraction and final prosthetic rehabilitation [1]. However, Araujo et al. [1–3] showed that the placement of immediate implants in a fresh extraction sites failed to prevent the post extraction bone remodeling that occurs along both buccal and lingual plates walls of the socket.

It was suggested that the resorption of the socket walls that occurs following tooth removal must be considered in any future treatment plan [4]. Various techniques have been proposed to treat class 2 socket types with immediate implant placement, the IDR, immediate dento-alveolar restoration that utilized the use of tuberosity bone graft introduced to the buccal defect incisally, thus restoring missing buccal bone walls. However, the technique lacks the stabilization of the graft to the host bed, the high remodeling rate of the tuberosity bone graft and the rare availability of the tuberosity bone when wisdom teeth exists. On the other hand, the early placement approach was heavily studied by Buser D. et al. [5, 6].

The technique entails the tooth extraction followed with a delay period of 8–12 weeks, the authors claimed that this period is allowed to develop abundant keratinized tissues, to eradicate socket infection, and to allow post extraction bone remodeling to occur. However, early placement or contour augmentation procedures has showed: post tooth extraction socket wall collapse in both horizonal and vertical directions, long treatment time that reaches up to 8 months, the hardship of provisional restoration maintenance for such long treatment time, post restorative socket tissue recession as a result of mucoperiosteal flap reflection [7]. Therefore, it is hard to predict a successful esthetic treatment outcome [8, 9].

The vestibular socket therapy allowed immediate implant placement and total socket rehabilitation at the same time, with a supreme esthetic and functional outcomes that meets patient’s expectations. Vestibular socket therapy (VST) was proposed by Elaskry et al. [10], where socket augmentation is carried out through minimally invasive vestibular access incision to allow the delivery of the grafting components with no need to perform the classic mucoperiosteal flap reflection regardless of the degree of socket compromise [10–12].

The technique entails a vestibular horizontal incision at the base of the mucogingival junction of the extracted tooth, followed by implant placement, grafting the defective socket walls via the vestibular access incision, then shielding the labial bone defect that is grafted with bone graft with cortical equine membrane. This study aims to compare the outcome and predictability of three grafting materials in treating the osseous defects in the maxillary anterior esthetic zone [11, 13].

## Methods

Twenty-four patients (17 females and 7 males) who presented with failed anterior bounded tooth the esthetic zone participated in this randomized controlled clinical trial. The subjects were recruited consecutively from the outpatient clinic of the Oral Medicine, Periodontology, Oral Diagnosis and Radiology Department at Faculty of Dentistry, Tanta University. The purpose of the study was explained to all patients, and informed consent was signed before the conduction of the study. The proposal was presented to the Faculty of Dentistry, Tanta University Research Ethics Committee and was approved before starting the research. (**OMPDR/04–21/13).** The procedures used in this study adhere to the tenets of the Declaration of Helsinki, and the study was registered on www.clinicaltrial.gov (**12/07/2021** - **ID: NCT04957654).**

### Eligibility criteria

**Fig. 1:**
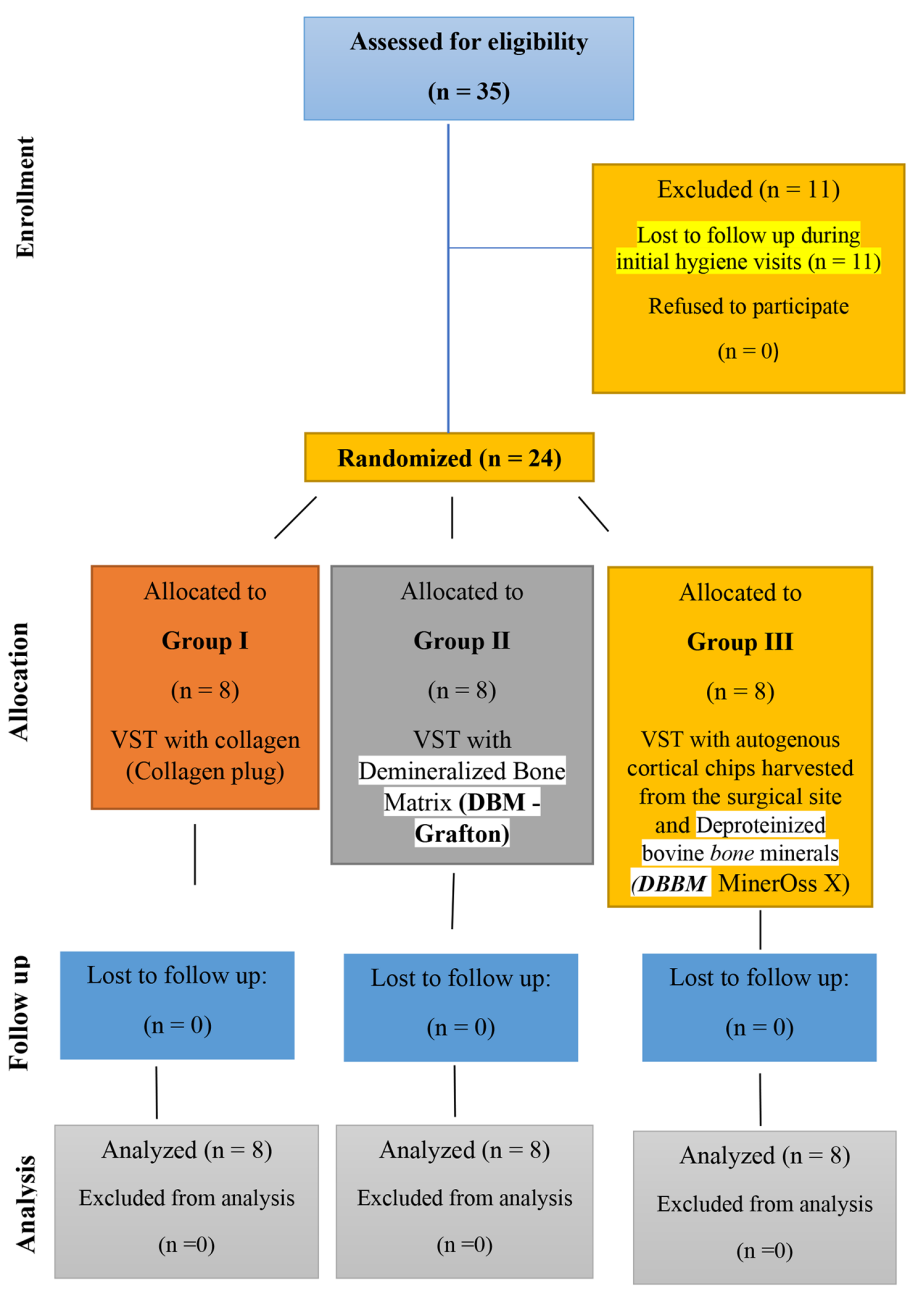
CONSORT flow diagram

Patients were selected according to the following main inclusion and exclusion criteria. Patients with one or more non-restorable teeth or remaining roots without signs of acute infection in the maxillary anterior region, sufficient bone (> 4 mm) apically and palatally to allow for proper implant positioning with sufficient primary stability (≥ 35 N cm), systemic free, and good compliance. The exclusion criteria included teeth with current acute periapical infection, medically compromised patients, heavy smokers, and vulnerable groups (pregnant females and decision-impaired individuals). **(**Fig. 1**)**

### Presurgical phase and treatment allocation

This study aimed to evaluate the esthetic, clinical, and radiographic outcomes following the placement of immediate implants with the vestibular socket technique in the esthetic zone. The number of patients in each group was determined by a sample size calculation and based on the results of the power analysis calculated by G-power 3.0.10 software to be twenty-one surgical sites. This was increased to twenty-four surgical sites to compensate for lost-to-follow-up cases. The twenty-four surgical sites were divided into three groups, each with eight surgical sites. Allocation concealment was achieved using sequentially numbered, opaque, sealed envelopes *(SNOSE)*. Preoperative cone beam computed tomography (CBCT) radiographs were obtained prior to immediate implant placement to confirm the socket type and for the construction of a computer-generated surgical guide. Then, intraoral scanning was performed on all patients to record mucosal levels and for assessment after implant placement. A full clinical examination (probing depth, attachment level, and bleeding index) was performed. Conventional periodontal treatment, including scaling and root planing was performed using ultrasonic and hand scalers and curettes, oral hygiene instructions were reiterated until patients achieved an optimal level.

**Materials**: A tapered pro implant platform switched design (Biohorizons, Birmingham, Al, USA) was used. The bone grafting material differed according to the treatment groups: group (I) used a Collagen plug, group (II) used a demineralized bone matrix **(DBM) Grafton**, and group (III) used a particulate bone graft composed of two-thirds autogenous cortical chips harvested from the surgical site and one-third deproteinized bovine bone minerals ***(DBBM)*** **MinerOss X** (Biohorizons, Birmingham, Al, USA) The graft material was overlaid by a flexible equine cortical membrane shield of 1 mm thickness (OsteoBiol® Lamina®, Technoss®, GiavenoTorino, Italy).

**Surgical procedure**: All surgical and restorative procedures were performed by a single investigator. Following oral rinse with 0.12% chlorhexidine HCl mouthwash (Hexitol, ADCO Pharma, Cairo, Egypt) and the administration of articaine HCl 4% with epinephrine 1:100 000 local anesthesia (Artinibsa 4%, Inibsa Dental S.L.U., Barcelona, Spain), a vestibular access horizontal incision was made using a 15-c blade (Stoma/Storz am Mark, Emmingen-Liptingen, Germany) at the socket site, located at the base of the vestibule, and extending horizontally to adjacent teeth. The tooth was atraumatically extracted followed by socket lavage and curettage.

The implant was delivered through the prefabricated surgical guide to ensure an optimum prosthetically driven implant position, with the implant shoulder placed 2 mm apical to the zeinth of the labial gingival margin and slightly palatal, and the graft well condensed to fill all the gaps between the implant and the extraction socket voids. A 1-mm-thick flexible cortical equine membrane of heterologous origin was trimmed and tacked through the vestibular access incision until it extended 1.0 mm below the gingival margin. It was then stabilized to the apical bone using two membrane tacks (AutoTac System Kit, Biohorizons, Birmingham, Al, USA).

The vestibular incision was then secured and sutured using 6/o polypropylene monofilament suture material, and then a customized healing abutment was used to seal the socket orifice thus isolating the bone graft components from the oral enviroment using composite resin (Filtek Supreme Ultra Flowable Restorative, 3 M, St Paul, MN, USA) and a temporary abutment (PEEK Temporary Cylinder, BioHorizons). Stutures were removed two weeks after surgery.

### Phase IV therapy (prosthetic phase)

Routine postoperative instructions were given to the patients in a written form. After 2 months, all patients were scanned for the fabrication of the final prosthesis. Then the definitive screw-retained zirconia-ceramic crowns were delivered [14]. CBCT was used for radiographic assessment preoperatively and at 6 and 12 months postoperatively.

**Outcomes measure**.

### Primary outcome: Pink Esthetic score (PES)

PES was assessed at 6 and 12 months postoperatively. Seven variables were scored: mesial papilla, distal papilla, curvature of the facial mucosa, level of the facial mucosa and soft tissue color, and texture of facial gingiva at the implant site. A score of 0, 1, or 2 was given to each parameter to obtain a final score of 14 [15].

### Secondary outcomes

A. **Soft Tissue Assessment – Peri-implant mucosal level (PML)**.

Intraoral scanning was performed at baseline and six months and twelve months postoperatively. The 3D software **(NemoSmile Design 3D, Nemotec, Madrid, Spain)** was used to align the pre- and six- and twelve-month models through three identical points using the best-fit algorithm of the software to perfect the superimposition process. The superimposed models were then imported into an STL viewer **(3Shape Ortho viewer, 3Shape, Denmark)**, where the measurements were performed. Three points were identified at the mesial papilla, mid-facial mucosa, and distal papilla, and for thickness changes, bucco-lingual soft tissue contour was measured at the crown margin in the mid-sagittal plane. Midfacial changes and mesial and distal papilla changes were measured as the linear difference between scans.

B. **Hard Tissue Assessment – Facial Bone Thickness (FBT)**.

Each group was subjected to cone beam computed tomography (CBCT) at baseline, six months, and twelve months to assess the thickness and height of the labial (facial) plate of bone and implant survival.

#### Statistical analysis

Data were collected and entered into the computer using the Statistical Package for Social Science (SPSS) program for statistical analysis (version 21) [10]. Data are presented as the mean and standard deviation (SD). The Kolmogorov‒Smirnov test of normality revealed significance in the distribution of most of the variables [12]. The results data were represented as a median since it is not normally distributed.

## Results

An original sample of 24 patients complied with the eligibility criteria with a survival rate of 100% in all groups. The age of the collagen plug group patients ranged from 35.00 to 55.00 years, with a median of 47.00 years and a 95% confidence interval (CI) of 45.00–55.00 years, while in the Grafton group, it ranged from 24.00 to 55.00 years, with a median of 40.50 years and a 95% CI of 25.00–46.00 years. In the MinorOssX group, the age ranged from 35.00 to 48.00 years with a median of 37.00 years, and the 95% CI was 36.00–48.00 years **(**Table 1**)**. Age was not significantly different among the three studied groups (*p* = .105). There was also no statistically significant difference between gender distributions in the two groups. All implants received screw-retained crowns.

**Table 1:** Comparison of age (years) among the three studied groups

Age (years)	Type of Bone graft		
	Collagen plug	Grafton	MinerOss X
- n	8	8	8
- Min. – Max.	35.00–55.00	24.00–55.00	35.00–48.00
- Median	47.00	40.50	37.00
- 95% CI of the median	45.00–55.00	25.00–46.00	36.00–48.00
**Test of significance** ***p***	*p* = .105		

n: Number of patients

Min-Max: Minimum – Maximum

CI: Confidence interval

KW = Kruskal‒Wallis H

*: Statistically significant (*p* < .05)

NS: Statistically not significant (*p* ≥ .05)

**Fig. 2:**
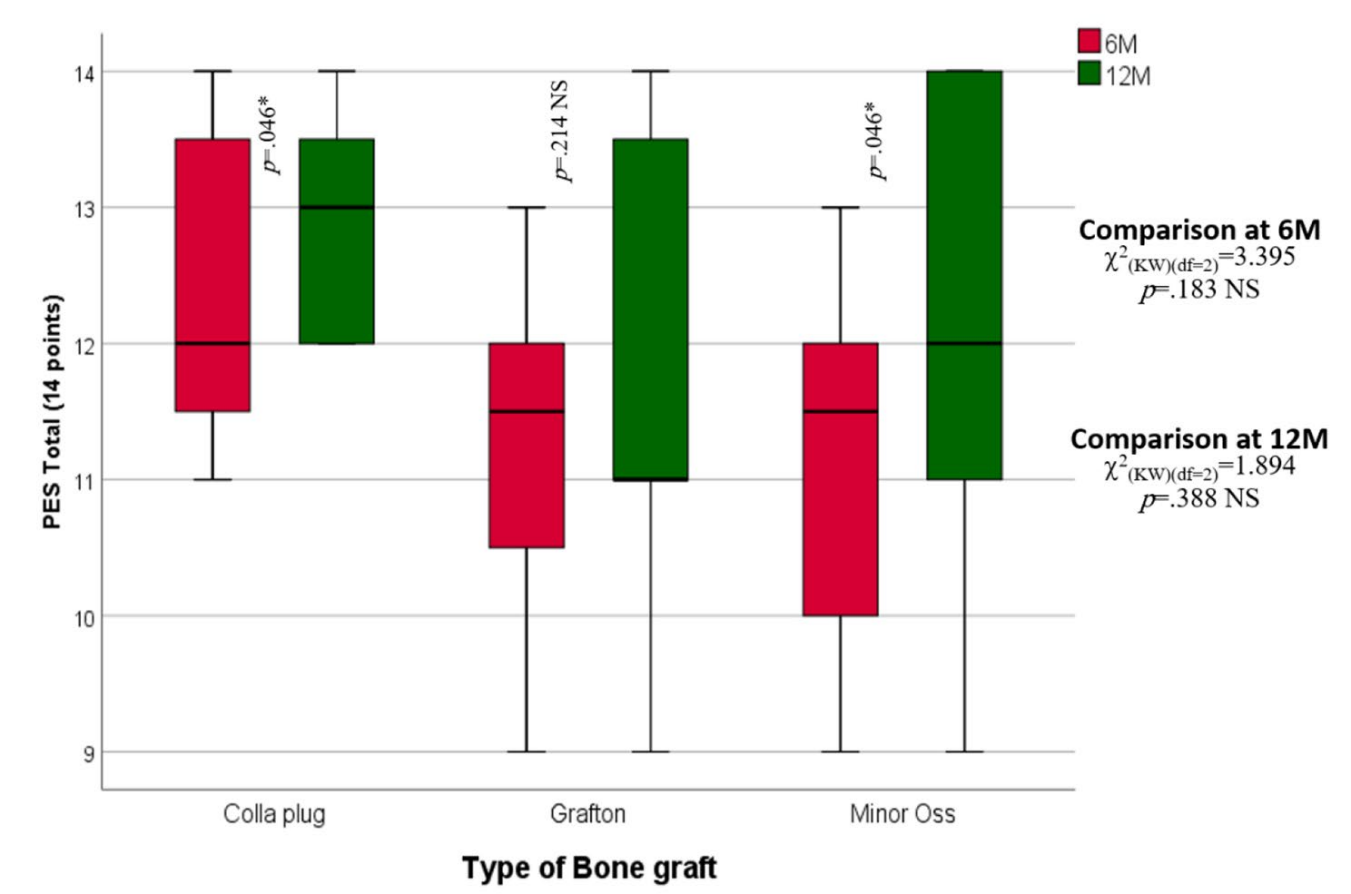
Box and whisker graph of PES in the studied groups. The thick line in the middle of the box represents the median, the box represents the interquartile range (from 25th to 75th percentiles), and the whiskers represent the minimum and maximum after excluding outliers (circles)

Regarding the median PES of the collagen plug group, the PES was 11.50 at 6 months and increased significantly to 12.00 at 12 months. In the Grafton group, the median PES was 11.50 at 6 months and showed a slight decline to 11.00 at 12 months, with no statistically significant difference. In the MinerOss X group, the PES was 12.00 at 6 months and increased significantly (*p* = .046) to 13.00 at 12 months. There was no statistical significance between the three groups, as shown in Table 2** (**Fig. 2**)**.

**Fig. 3:**
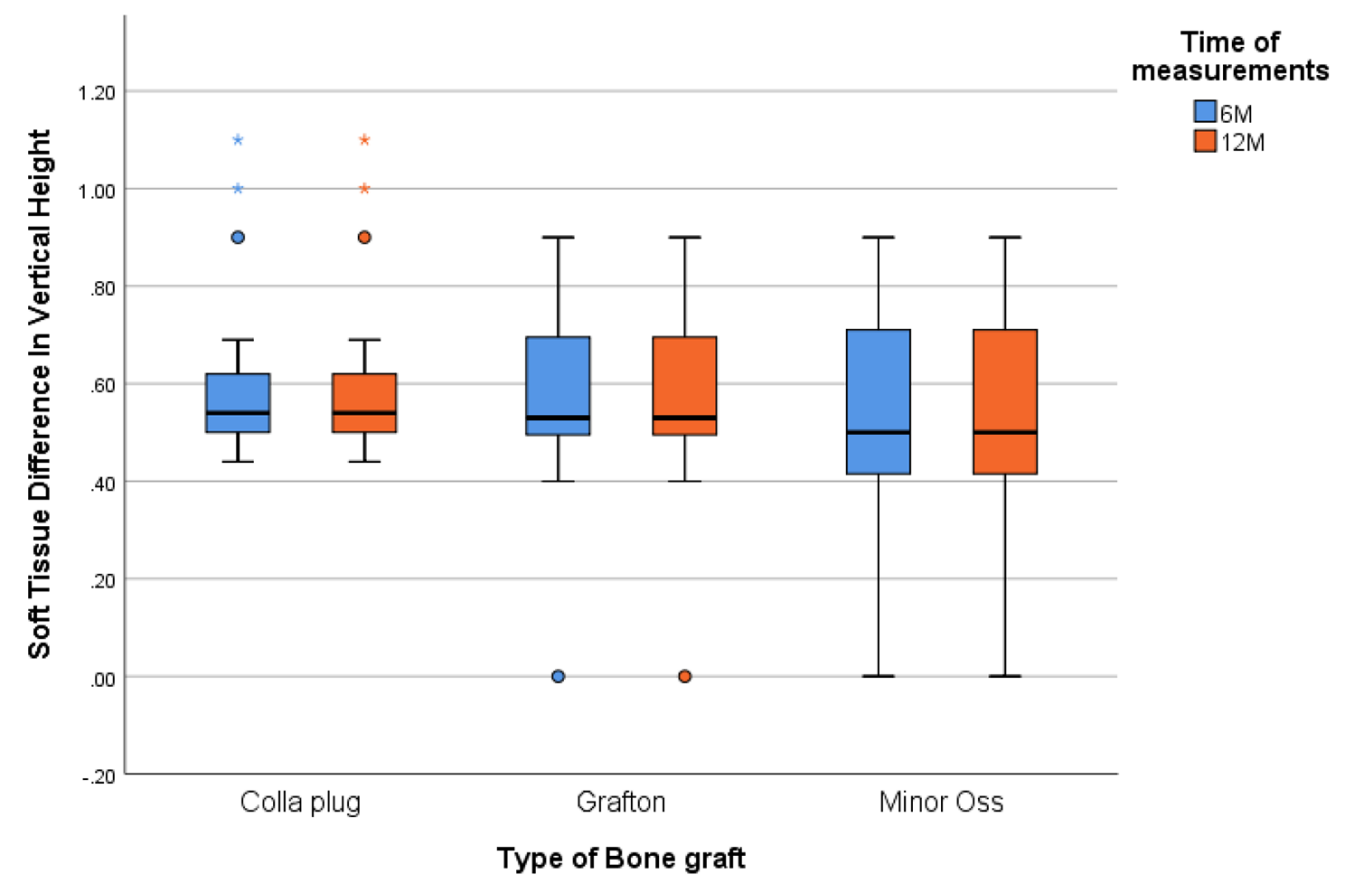
Box and whisker graph of mean soft tissue (mm) in the studied groups. The thick line in the middle of the box represents the median, the box represents the interquartile range (from 25th to 75th percentiles), the whiskers represent the minimum, and the maximum represents the minimum and maximum after excluding outliers (circles)

At six months, the median of the soft tissue difference in vertical height (mm) of the collagen plug was 0.55 mm, while in the Grafton group, it was 0.58 mm, and in the MinerOss group, it was 0.57 mm. These results were maintained until the end of the study period (12 months). There was no statistically significant difference among the three studied groups at six months (*p* = .300). Table 2** (**Fig. 3**)**.

**Fig. 4:**
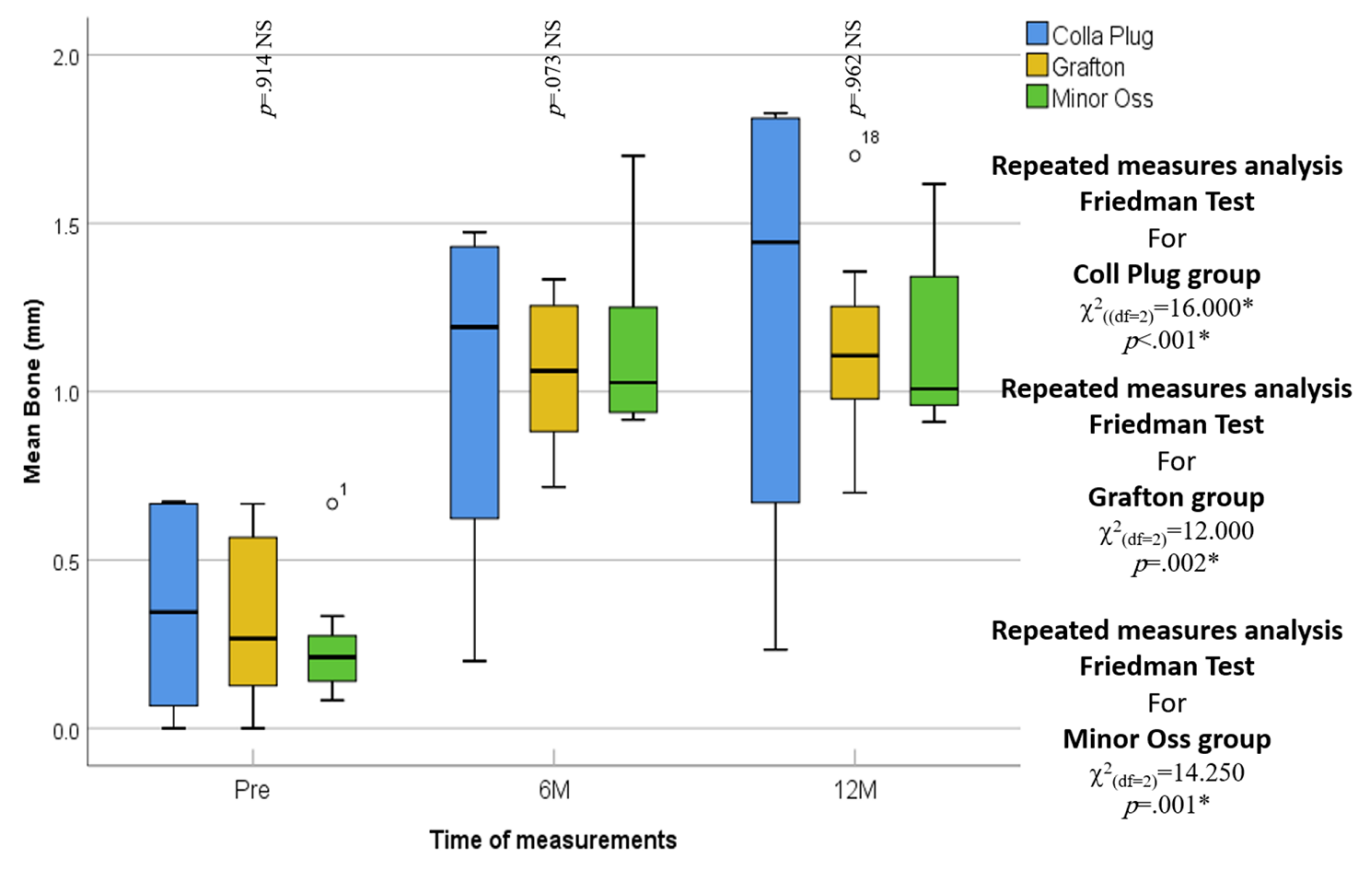
Box and whisker graph of mean bone (mm) in the studied groups. The thick line in the middle of the box represents the median, the box represents the interquartile range (from 25th to 75th percentiles), the whiskers represent the minimum, and the maximum represents the minimum and maximum after excluding outliers (circles)

The median radiographic facial bone thickness for the collagen plug group was 0.21, which significantly increased to 1.03 and 1.01 at 6 and 12 months, respectively (*p* = .008). The same was true regarding the Grafton and MinorOss X groups. The FBT was 0.27 and 0.35 at baseline, respectively, which increased significantly in the Grafton group to 1.06 and 1.11 mm at 6 months and 12 months, respectively (*p* = .024). Additionally, for the Minor Oss group, it increased to 1.19 and 1.44 mm at 6 and 12 months, respectively, with a statistically significant difference when compared to baseline (p 0.027). However, there was no statistically significant difference among the three studied groups at six (*p* = .898) and twelve months (*p* = .523). **(**Table 3**) (**Fig. 4**)**.

**Table 2:** Inter- and intragroup comparisons of the Collagen plug, Grafton, and MinerOss X groups regarding the pink esthetic score and soft tissue difference in vertical height at 6 and 12 months postoperatively

	Pink Esthetic Score (PES) (Mean ± SD)			
Interval	Collagen plug	Grafton	MinerOss X	P value
PES Total (6 M)				*p* = .183
- n	8	8	8	
- Min. – Max.	9.00–13.00	9.00–13.00	11.00–14.00	
- Median	11.50	11.50	12.00	
95% CI of the median	10.00–12.00	10.00–12.00	12.00–14.00	
PES Total (12 M) - n	8	8	8	*p* = .388
- Min. – Max.	9.00–14.00	9.00–14.00	12.00–14.00	
- Median	12.00	11.00	13.00	
95% CI of the median	11.00–14.00	11.00–14.00	13.00–14.00	
Test of significance *p*	Z_(WSR)_ = 1.994 *p* = .046*	Z_(WSR)_ = 1.242 *p* = .214 NS	Z_(WSR)_ = 2.000 *p* = .046*	
	**Soft Tissue Difference in Vertical Height** (Mean ± SD)			
Six Months				*p* = .892
- n	8	8	8	
- Min. – Max.	0.52–0.87	0.41–0.63	0.30–0.69	
- Median	0.55	0.58	0.57	
95% CI of the median	0.52–0.70	0.57–0.63	0.38–0.68	
Twelve Months				*p* = .892
- n	8	8	8	
- Min. – Max.	0.52–0.87	0.41–0.63	0.30–0.69	
- Median	0.55	0.58	0.57	
95% CI of the median	0.52–0.70	0.57–0.63	0.38–0.68	
P value	NA Due to exact match	NA Due to exact match	NA Due to exact match	

n: Number of patients

Min-Max: Minimum – Maximum

CI: Confidence interval

KW = Kruskal‒Wallis H

*: Statistically significant (*p* < .05)

NS: Statistically not significant (*p* ≥ .05)

**Table 3:** Inter- and intragroup comparisons of Groups I, II and III regarding facial bone thickness preoperatively and 6 and 12 months postoperatively

	Facial Bone Thickness (FBT) (Mean ± SD)			
Interval	Collagen plug	Grafton	MinerOss X	P value
Pre				*p* = .990
- n	8	8	8	
- Min. – Max.	0.08–0.67	0.00-0.67	0.00-0.67	
- Median	0.21	0.27	0.35	
95% CI of the median	0.13–0.33	0.10–0.63	0.07–0.67	
Six Months				*p* = .898
- n	8	8	8	
- Min. – Max.	0.92–1.70	0.72–1.33	0.20–1.47	
- Median	1.03	1.06	1.19	
95% CI of the median	0.92–1.40	0.78–1.32	0.27–1.46	
Twelve Months				*p* = .523
- n	8	8	8	
- Min. – Max.	0.91–1.62	0.70–1.70	0.23–1.83	
- Median	1.01	1.11	1.44	
95% CI of the median	0.95–1.58	0.89–1.36	0.31–1.82	
Friedman Test	c^2^_(df=2)_ = 9.600	c^2^_(df=2)_ = 7.467	c^2^_(df=2)_ = 7.200	
*p*	*p* = .008*	*p* = .024*	*p* = .027*	

n: Number of patients

Min-Max: Minimum – Maximum

S.D.: Standard deviation

CI: Confidence interval

*p*: Probability of error (chance)

KS = Kolmogorov‒Smirnov

KW = Kruskal‒Wallis H

WSR = Wilcoxon Signed Rank Test

*: Statistically significant (p < .05)

NS: Statistically not significant (p > .05)

## Discussion

An optimum treatment outcome should be presented to the patient specifically in the maxillary esthetic region where immediate implants are considered. Post restorative bone remodeling followed with mid facial post restorative gingival recession should be taken into consideration. To date, several techniques have been developed to counteract this inevitable bone loss [16, 17]. Elaskry et al. [12], in 2020, developed the “vestibular socket therapy”, for treating a wide variety of fresh extraction sockets with an intact, thin, or even totally lost labial plate of bone with or without signs of active infection. The VST preserved peri-implant soft tissues by implementing the vestibular access incision which has led to a remarkable stability of the soft tissue margins. The aim of this current work was to offer the clinician the optimal graft recipe to use along with the VST protocol.

The use of platform switched dental implants maintained crestal bone levels, and enhanced the peri-implant tissue stability [18]. The three bone grafting components used in this study showed various results; the collagen plug group, seemed not to perform as expected because of the lack of structural support to the overlying cortical membrane, while the grafton group showed a more enhanced regeneration because of its osteoinductive properties, as being a demineralized bone matrix (DBM) manufactured with aseptic processing technology that preserves the function of naturally occurring growth factors that provided a high osteoinductivity score [19–21].

By using particulated bone graft in the MinerOss X group, the autogenous cortical bone chips offered a sustained release soluble signals that can modulate differentiation of mesenchymal cells in vitro involving TGF-β1 signaling within 10 min & Bmp-2 in 40 min that allowed the biological activity of the bone graft and induced the activation of the osteogenic properties of bone graft components [22–25].

Combining the xenogeneic bone graft particles to the particulated bone graft assisted in providing a stable graft matrix due to its slow remodeling speed [26–29]. It also provided space maintenance and a more favorable physical environment for regeneration.

In the collagen plug group, there was a significant decrease in the overall thickness (1.01 mm) of the labial bone after 12 months compared to the Grafton and MinerOss X groups (1.11 & 1.44 mm). That was in line with a previous published work by Bakkali et al. and Novaes et al. revealed that grafting coupled with implant placement significantly reduced horizontal bone resorption and showed superior outcomes compared to the reliance on blood clot reorganization alone [30, 31].

Nonetheless, due to the small sample size of the present study, the differences were not statistically significant. In the current study, patients with thin soft tissue phenotypes were excluded to avoid confounding results [32, 33].

This study showed that the vestibular socket therapy protocol is predictable in maintaining high esthetic outcome in all groups in class II fresh extraction sockets with immediate implant placement. Soft tissue vertical height was maintained for all sockets included in the present study and showed a successful outcome at the end of the study period (12 months). The same rates were observed in a recently published prospective 2-year follow-up clinical studies with provided evidence for long-term stability of both bone and soft tissue architectures with predictable radiographic and esthetic outcomes advocating the use of the VST for immediate implant placement in class II fresh extraction sockets with or without signs of infection [11, 12].

Using a slow biodegradable and flexible cortical xenograft shield along with VST provided support to the overlying mucosa, minimized postoperative tissue collapse, allowed the labial bone plate to undergo the resorption phase followed by bone regeneration with no dimensional changes in the socket size, and protected the labial gap from epithelial invasion [11, 12]. The use of the cortical membrane bypassed the deleterious effect of both post extraction bone remodeling [34], and graft resorption through its long-term biodegradation rate.

It is worth noting that all implants were positioned at 2– 3 mm below the zenith of the mid-facial gingival point to provide a proper emergence profile and establish an optimal biological width with the regenerated bone de-nuvo. Additionally, a sufficient gap between the implant and the facial bone was left for the graft materials or the blood clot to increase the regenerative space. This allowed more room for regeneration, thus providing a thicker buccal bone to be regenerated [35, 36].

Furthermore, customized healing abutments used at the same time of immediate implant placement preserved the socket architecture during treatment phases, sealed the socket environment from oral bacteria, and minimize the prosthetic soft tissue profiling needed in the restorative phase [37].

The currently presented findings demonstrated that the overall PES values after 12 months were 12.00 in group I (collagen plug), while group II (Grafton) showed scores of 11.00 and group III (MinerOss X) showed scores of 13.00, without a statistically significant difference observed between the studied groups. This suggested that optimum esthetics were achieved when the vestibular socket protocol for immediate implant placement was performed. Similarly, Elaskry and coworkers [10, 11] observed satisfying esthetic outcomes with good PES scores (11.33 and 12.63, respectively) after using vestibular socket therapy for treating fenestrated and compromised fresh extraction sockets with immediate implants. The improvements in PES and FBT can be attributed to the nature of the VST technique, which utilizes a conservative, minimally invasive, and biologically driven approach to managing socket related tissues. Using a single vestibular incision preserved the marginal mucosal levels, which in turn enhanced the esthetic outcome, and minimized post extraction recession via minimizing the osteoclastic activities around the socket margins [38–42].

Immediate implant placement offers the benefits of reducing patient visits and preserving alveolar bone and its related soft tissues. The short-lived satisfaction is then followed by compromised esthetics and patient dissatisfaction resulting from possible post-restorative long term marginal recession and crestal bone loss. To date, there is no single technique or treatment regimen with substantiating evidence to address all clinical scenarios with long-term stability. However, with the use of VST along with biologically active bone grafting components, VST has shown evidence of a superior treatment outcome in treating hopeless maxillary anterior failed dentition.

The limitations of this study remains in the small sample size of the groups and the inapplicability of the technique for use in failed posterior teeth. Emphasis should be placed on adequately filling the socket defects and the jumping gap with bone graft components to achieve an optimal regeneration of the lost labial plate of bone [13].

## Conclusions

Within the limitations of this study in comparing different graft materials used with the VST, the particulate bone graft showed enhanced regenerative outcomes when compared with blood clot or Grafton. The particulate grafts could be offered as a predictable regenerative option in treating defective socket osseous walls. The VST could be considered as a gold standard in treating class 2 fresh extraction sites in the esthetic zone with high predictability. However, further randomized clinical trials with longer follow-up intervals and a larger sample size are required to validate these observations.

## Data Availability

The datasets used and/or analyzed during the current study are available from the corresponding author upon reasonable request.

## Declarations

### Protocol availability

The full trial protocol is available and can be accessed in the related file section.

## Abbreviations

• **VST**: • Vestibular Socket Therapy
• **FBT**: • Facial Bone Thickness
• **PML**: • Perimplant Mucosal Level
• **PES**: • Pink Esthetic Score
• **DBM**: • Demineralized Bone Matrix
• **DBBM**: • Deproteinized Bovine Bone Mineral
